# Supplementary material for: Unsupervised full-color cellular image reconstruction through disordered optical fiber
Source: Light Sci Appl. 2023 May 23;12:125. doi: 10.1038/s41377-023-01183-6 (PMC10206102; doi:10.1038/s41377-023-01183-6)
Supplement: Supplementary file 1 — Supplementary Information [file 41377_2023_1183_MOESM1_ESM.docx]

**Supplementary Information for**

**Unsupervised Full-color Cellular Image Reconstruction through Disordered Optical Fiber**

Xiaowen Hu^1^, Jian Zhao^2,*^, Jose Enrique Antonio-Lopez^1^, Rodrigo Amezcua Correa^1^, and Axel Schülzgen^1^

^1^CREOL, The College of Optics and Photonics, University of Central Florida, Orlando, FL 32816, USA

^2^The Picower Institute for Learning and Memory, Massachusetts Institute of Technology, Cambridge, Massachusetts 02139, USA

*Corresponding authors: [jianzhao@knights.ucf.edu](mailto:jianzhao@knights.ucf.edu)

**Imaging under low-light conditions**

**
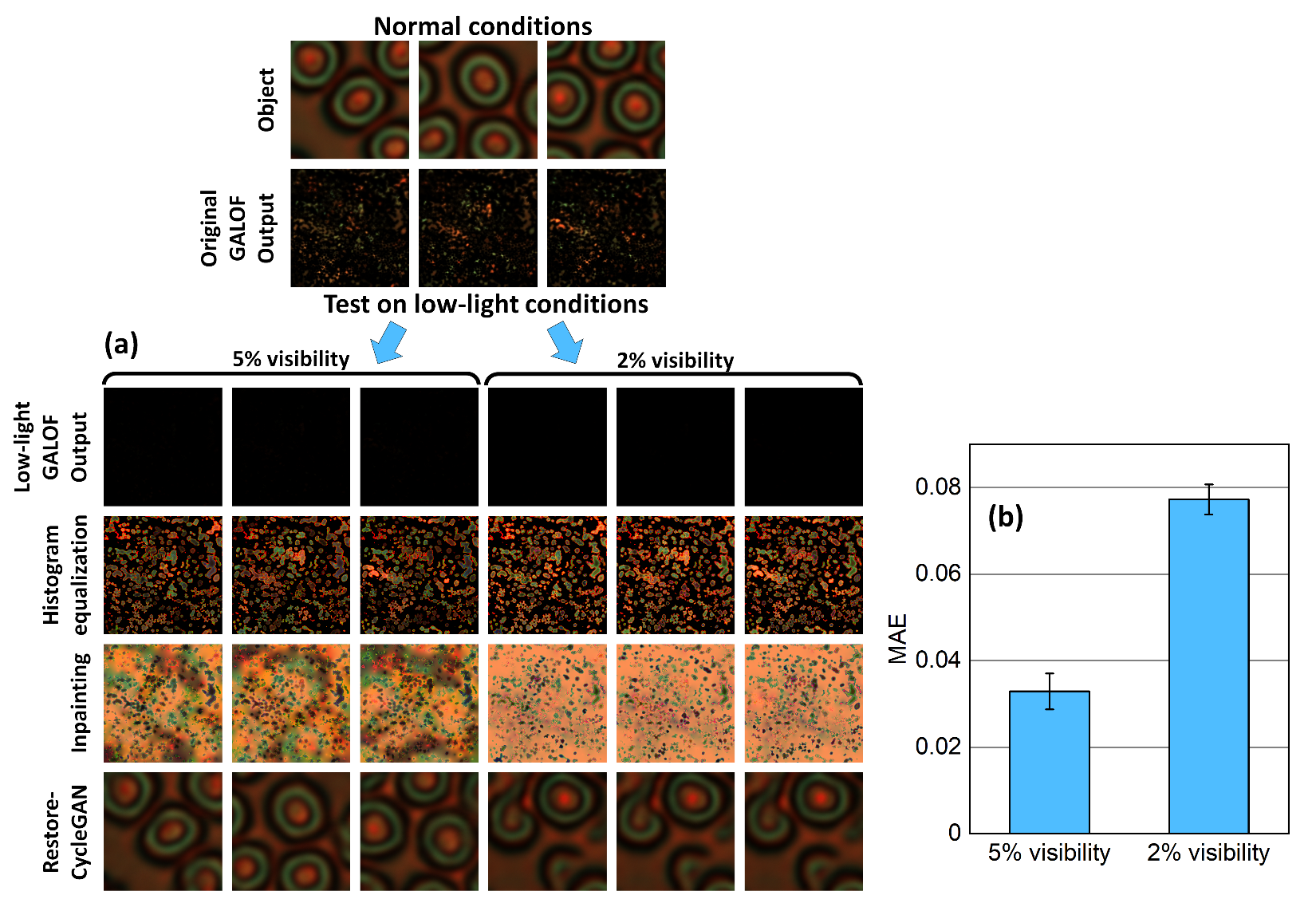
**

**Fig. S1** Numerical simulations of unsupervised image reconstruction under low-light conditions. (a) Sample images of the objects, the original GALOF outputs, the low-light GALOF outputs with 5 % visibility and 2 % visibility, the pre-processed images (excluding the registration step), and the final reconstructed images. (b) MAE averaged over 1,000 reconstructions. The error bar stands for STD.

We numerically simulate low-light imaging using the images collected for a straight GALOF with a working distance of 0 mm. Under normal illumination, the pixel values of the original GALOF output range from 0 to 255 for an 8-bit image. In our low-light imaging simulation, the pixel value ranges of the GALOF outputs are reduced to 0-13 and 0- 5 for 8-bit images, corresponding to 5 % and 2 % visibility, respectively. Due to the low-light level, the previous criteria for defective pixels, specifically the maximum value being less than 10 or the standard deviation (STD) being less than 2, is no longer applicable. Instead, we adopt the maps of the defective pixels measured from the original GALOF outputs, as the defective pixels are intrinsic in the fiber and can be easily characterized. Besides that, we follow the same calibration procedure presented in the manuscript. **Fig. S1a** shows the images after each pre-processing and reconstruction step. Due to more restricted pixel value ranges in low-light conditions, the images processed by the histogram equalization step and the inpainting step exhibit greater blurriness. Nevertheless, the profiles of the human red blood cells are still recognizable under 5 % visibility. High-fidelity images are then reconstructed by a Restore-CycleGAN, showing an excellent low-light imaging capability. For even lower visibility of 2 %, the processed images after the inpainting step are hardly recognizable. As a result, the Restore-CycleGAN runs into the “mode collapse”^1^, where identical reconstructions are generated regardless of the inputs. The mean absolute errors (MAEs) and the STDs of the 1,000 test reconstructions are shown in **Fig. S1b**.

**Imaging under high-noise levels**

**
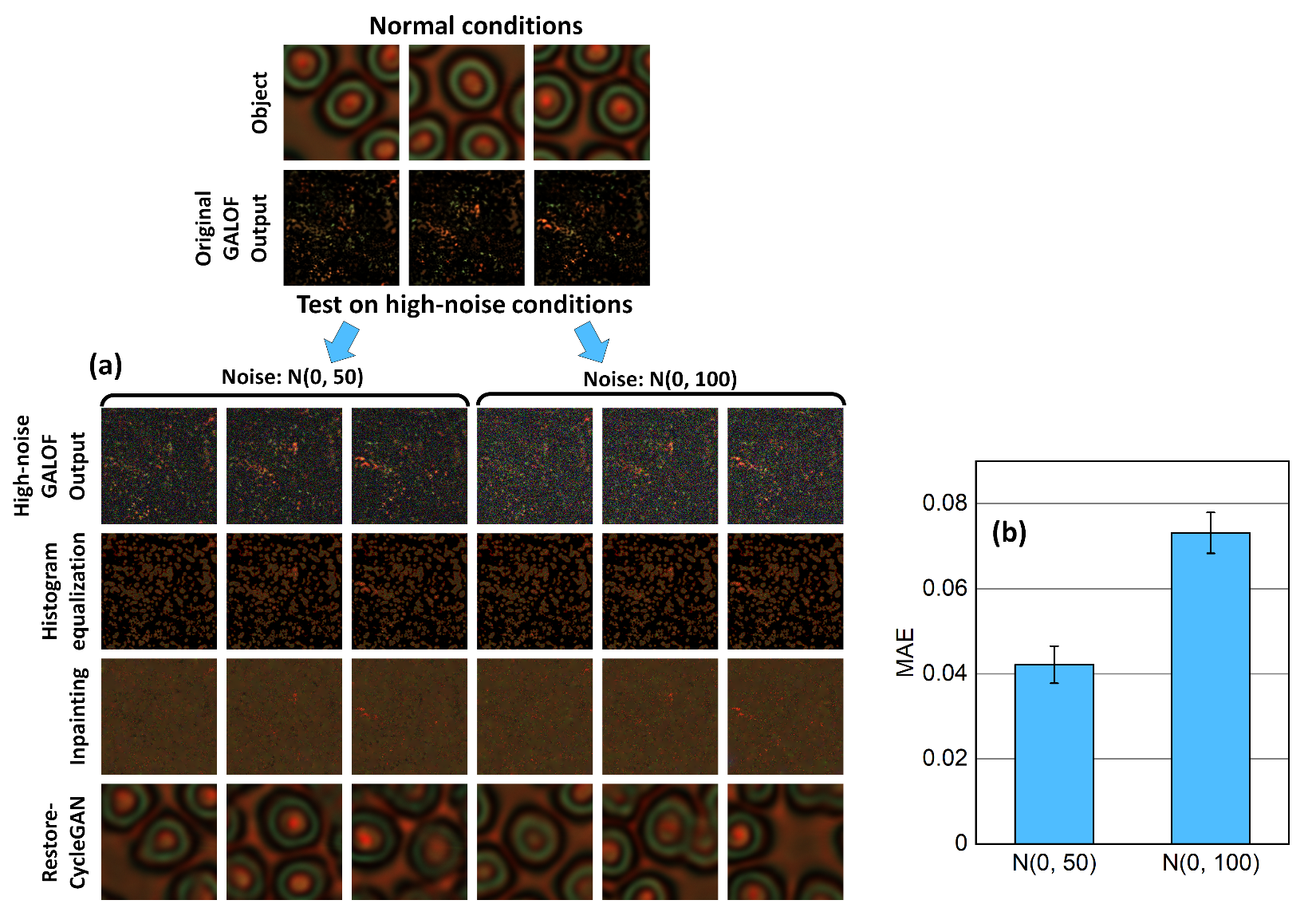
**

**Fig. S2** Numerical simulations of unsupervised image reconstruction under high-noise conditions. (a) Sample images of the objects, the original GALOF outputs, the high-noise GALOF outputs with Gaussian noises of *N*(0, 50) and *N*(0,100), the pre-processed images (excluding the registration step), and the final reconstructed images. (b) MAE averaged over 1,000 reconstructions. The error bar stands for STD.

To numerically test the unsupervised imaging with high noise levels, we introduce Gaussian noises to each RGB channel of the GALOF original outputs obtained from a straight fiber with a working distance of 0 mm. Specifically, we simulate two types of Gaussian noise distributions: *N*(0, 50) and *N*(0, 100). Both distributions have a mean value of 0, but their variance values are 50 and 100, respectively. The pixel values in the GALOF outputs range between 0~255 for the 8-bit image. We follow the same calibration procedure presented in the manuscript except for using the maps of the defective pixels measured from the original GALOF outputs. As shown in **Fig. S2a**, the reconstructions still demonstrate high fidelity under the *N*(0, 50) Gaussian noise, showing extraordinary resistance to noises. However, a larger variance of 100 tends to obscure the statistical information of the GALOF outputs, resulting in low-fidelity reconstructions. The corresponding MAEs and STDs of the 1,000 test reconstructions are plotted in **Fig. S2b**.

**Imaging under uneven illuminations**

We numerically simulate imaging under uneven illumination by multiplying the reference objects and the GALOF outputs with Gaussian functions. The Gaussian functions have maximum values of 1. The centers of the Gaussian functions are (210, 210) for the GALOF output and (180, 270) for the reference object, respectively, while the Gaussian radius is 210, with all parameters based on pixel counts (image size: 420×420). The GALOF outputs are obtained from a straight fiber with a working distance of 0 mm. Since uneven illumination changes the distribution of the pixel values in the reference objects, a histogram equalization on the reference objects (**Fig. S3a**) should be performed. Referring to **Fig. S3**, each pixel has a range of 1,000 values varying between 0 and 255. Similar to **Fig. 7** in **Methods**, we compute the Probability Mass Functions (PMFs) of the *N*×*N* pixels from the 1,000 reference objects, resulting in *N*×*N* PMFs (probability versus pixel value). To determine the reference PMF, we calculate the maximum and the STD for pixel value distribution. By comparing maxima from different pixel value distributions, we select the PMF of the pixel with the largest maximum value. In cases where two or more pixel value distributions share the same maximum value, we further compare their STDs and choose the PMF with the largest STD as the reference. The PMFs are then converted to Cumulative Distribution Functions (CDFs). We further compare the CDF of the reference pixel with the CDFs of all the other pixels for each pixel value to determine the mapping relation for identical CDF values (**Fig. 7b** in **Methods**). The Look-Up Tables (LUTs) are created based on these mappings to correct uneven illuminations (**Fig. S3a**). After the uneven illumination corrections in the reference objects, we follow the same method (**Principles**) to pre-process and reconstruct the images. **Fig. S3b** shows sample images of the reference objects alongside the GALOF outputs before and after multiplication by the Gaussian distributions, as well as the processed images following each pre-processing and reconstruction step. As illustrated in **Fig. S3b**, the reconstructions of high-fidelity images are achieved, with MAEs comparable to those obtained under uniform illuminations, as shown in **Fig. S3c**. These results demonstrate the remarkable robustness of unsupervised image reconstruction to uneven illuminations.


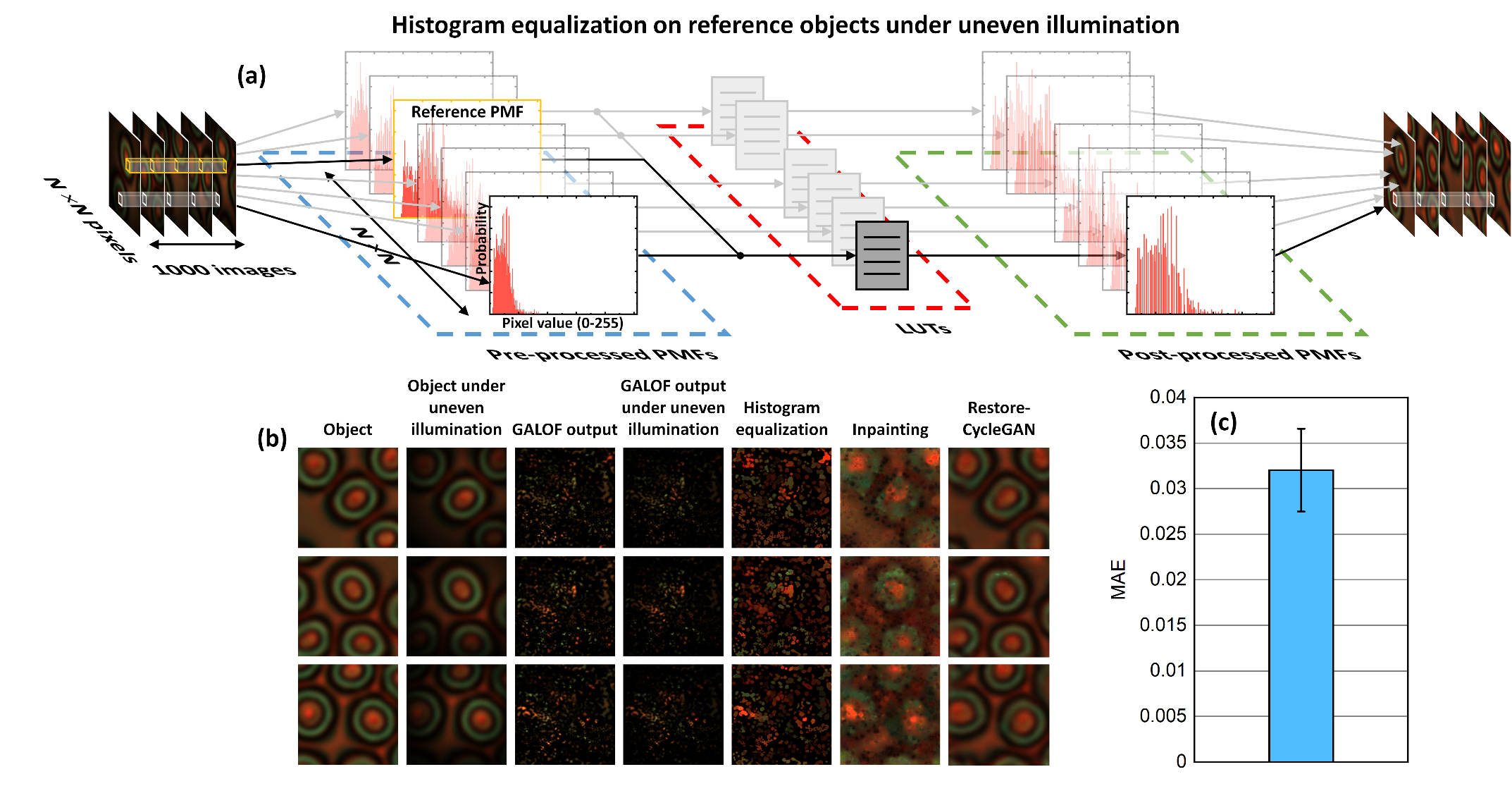


**Fig. S3** Numerical simulations of unsupervised image reconstruction under uneven illumination. (a) Histogram equalization for pixels in reference objects. For simplicity, only the red channel is shown. (b) Sample images of the original reference objects and GALOF outputs, the reference objects and GALOF outputs under uneven illumination, and the processed images after each reconstruction step (excluding the registration step). (c) MAE averaged over 1,000 reconstructions. The error bar stands for STD.

**Step-by-step analysis of unsupervised image reconstruction**

In **Fig. S4a**, we plot the MAEs and STDs of the processed images at each pre-processing and reconstruction step (the blue columns) for a straight GALOF with a working distance of 0 mm. Based on the data presented in **Fig. S4**, each pre-processing step improves the imaging quality, bringing it closer to the ground truths. This improvement enables the Restore-CycleGAN to find a natural translation for the final image reconstruction (the yellow arrow in **Fig. S4a**). The necessity of pre-processing is evident when we perform a Restore-CycleGAN reconstruction directly using un-processed raw GALOF outputs (the green arrow in **Fig. S4a**). In this scenario, the Restore-CycleGAN fails to recognize the features of the GALOF outputs and produces identical reconstructions (**Fig. S4b**) with high MAEs (the red column in **Fig. S4a**), indicating mode collapse^1^.


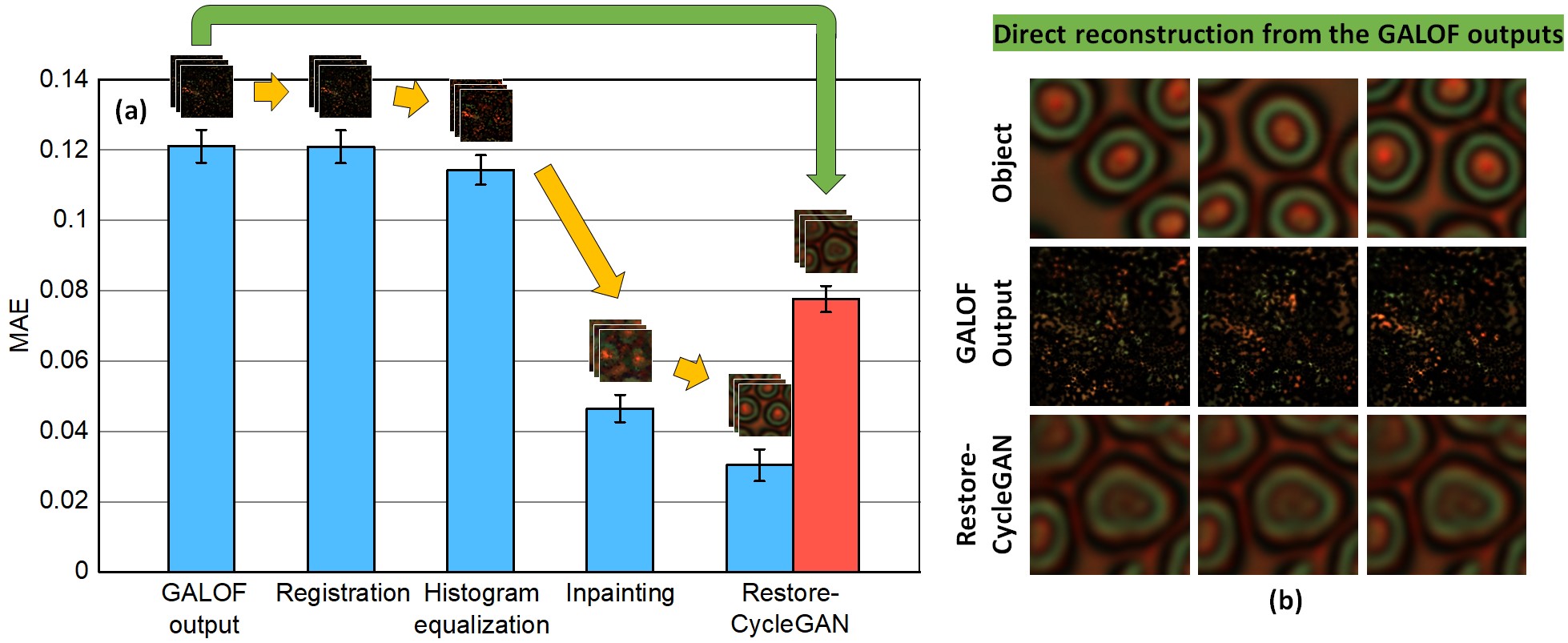


**Fig. S4** Step-by-step analysis of image reconstructions. (a) Blue column/Yellow arrow: the MAEs and STDs of pre-processed images and the final reconstructions; Red column/Green arrow: the MAEs and STDs of the final reconstructions without pre-processing. (b) Sample images of the objects, the GALOF outputs, and the reconstructions without pre-processing (Red column/Green arrow in a).

**Confidence metric for image reconstruction**


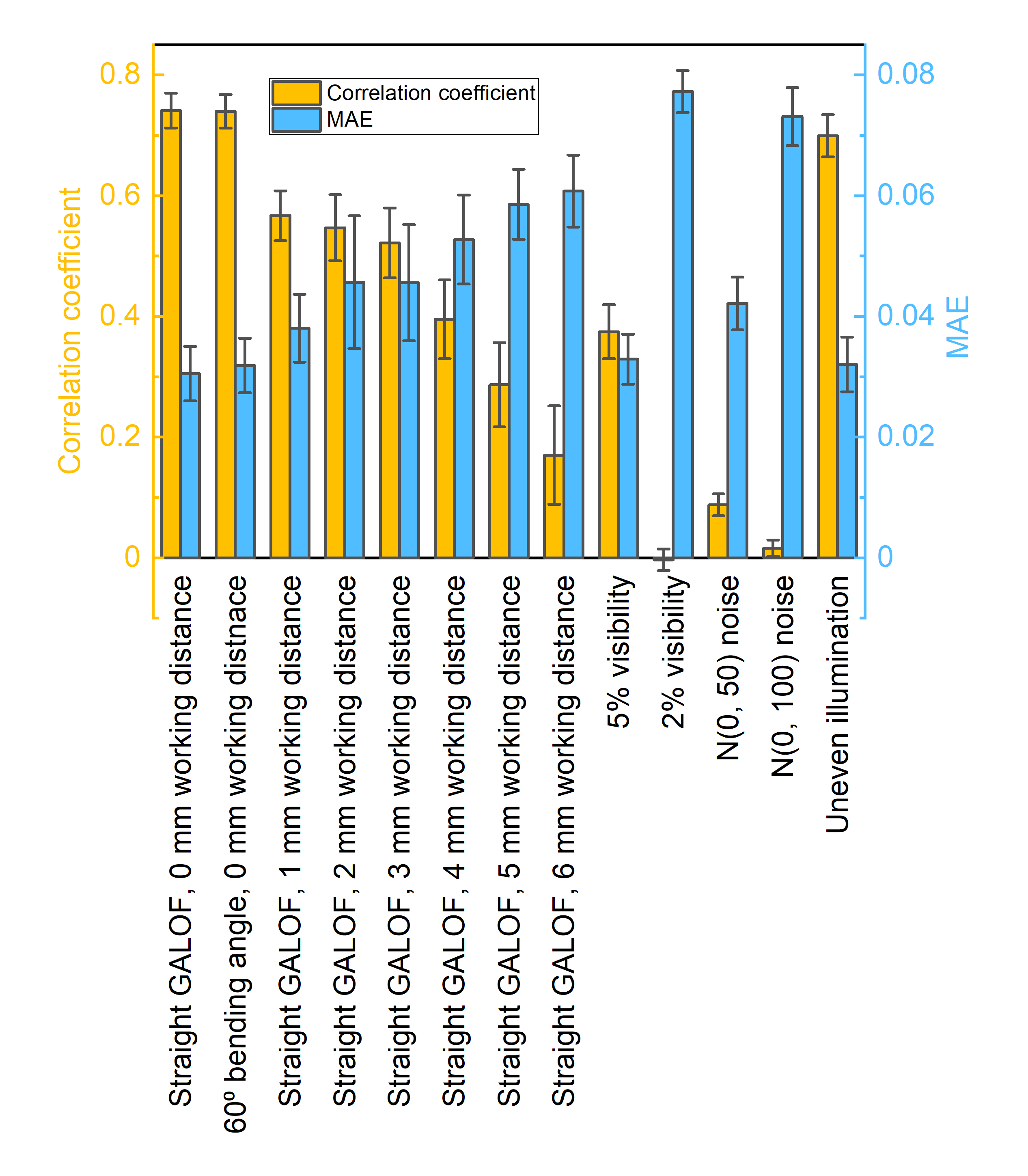


**Fig. S5** A comparison between the correlation coefficients and the MAEs for imaging under different configurations. Yellow columns: the correlation coefficients between pre- and post-processed images by Restore-CycleGAN. Blue columns: the MAEs between the reconstructions and the ground truths. Sample: human red blood cells.

The function of a confidence metric is to evaluate the fidelity of the image reconstruction without the ground truth. Here, we propose the correlation coefficient between pre- and post-processed images by the Restore-CycleGAN as the confidence metric. We first convert the RGB images to grayscale images using the MATLAB “*rgb2gray*” function. Then we calculate the correlation coefficient by

(1)

where $i,j$ are the indices of the pixels in the 2D images $A$ and $B$. $\overline{A}$ and $\overline{B}$ are the mean pixel values of $A$ and $B$, respectively. In **Fig. S5**, we plot the mean correlation coefficients (the yellow columns) and the MAEs (the blue columns) for the image reconstructions under various conditions in this work. The MAEs are calculated by comparing the reconstructions with their corresponding ground truths. In most cases, large correlation coefficients are consistent with the small MAEs, which shows the validity of the correlation coefficient as a confidence metric. Yet, correlation coefficients tend to be sensitive to high noise levels, as shown by a steep drop for a high level of Gaussian noise (**Fig. S5**), even when the MAE still indicates reasonably well imaging quality. Nevertheless, the relative degree of confidence is well preserved between different levels of Gaussian noise of *N*(0, 50) and *N*(0, 100).

Supplementary information accompanies the manuscript on the Light: Science & Applications website ([http://www.nature.com/lsa](https://nam02.safelinks.protection.outlook.com/?url=http%3A%2F%2Fwww.nature.com%2Flsa&data=05%7C01%7Cjianzhao%40knights.ucf.edu%7Ce96a8d7888044f764af708db5294e0d0%7C5b16e18278b3412c919668342689eeb7%7C0%7C1%7C638194571638243688%7CUnknown%7CTWFpbGZsb3d8eyJWIjoiMC4wLjAwMDAiLCJQIjoiV2luMzIiLCJBTiI6Ik1haWwiLCJXVCI6Mn0%3D%7C3000%7C%7C%7C&sdata=a1TZLgYO9ya%2BCTL%2F8GhhM3Xond7M%2FIi4KqB8ooYdCKQ%3D&reserved=0)).

**References**

1. Goodfellow I. Nips 2016 tutorial: Generative adversarial networks. *arXiv preprint arXiv:170100160*, (2016).
